# MIF-Associated Immunosuppressive CAF Remodeling Predicts Poor Prognosis During Lung Adenocarcinoma Progression: A Single-Cell and Multicohort Transcriptomic Study

**DOI:** 10.3390/biomedicines14071581

**Published:** 2026-07-15

**Authors:** Guo Lin, Jianrui Ji, Fan Ge, Zhouguang Hui

**Affiliations:** 1 Department of Radiation Oncology, National Cancer Center/National Clinical Research Center for Cancer/Cancer Hospital, Chinese Academy of Medical Sciences and Peking Union Medical College, Beijing 100021, China; 2 Department of Thoracic Surgery, National Cancer Center/National Clinical Research Center for Cancer/Cancer Hospital, Chinese Academy of Medical Sciences and Peking Union Medical College, Beijing 100021, China; 3 Department of VIP Medical Services, National Cancer Center/National Clinical Research Center for Cancer/Cancer Hospital, Chinese Academy of Medical Sciences and Peking Union Medical College, Beijing 100021, China

**Keywords:** lung adenocarcinoma, cancer-associated fibroblast, single-cell RNA sequencing, machine learning, tumor microenvironment

## Abstract

**Background:** Lung adenocarcinoma (LUAD) develops through a stepwise pathological status from atypical adenomatous hyperplasia (AAH), adenocarcinoma in situ (AIS), and minimally invasive adenocarcinoma (MIA) to invasive adenocarcinoma (IA). Although malignant epithelial evolution during this process has been increasingly characterized, the dynamic remodeling of cancer-associated fibroblasts (CAFs) and their contribution to the immunosuppressive tumor microenvironment (TME) remain incompletely explored. **Methods:** Single-cell RNA sequencing data from treatment-naïve LUAD lesions, including AAH, AIS, MIA, and IA, were analyzed together with external bulk transcriptomic cohorts. CAF subsets were characterized according to their transcriptional features, inferred developmental states, transcription factor activity, functional programs, and predicted cell–cell interactions. Ligand–receptor analysis was used to examine MIF-related communication between epithelial cells and CAFs. MIF-related genes were then used to develop a machine learning-based prognostic signature in the TCGA-LUAD cohort, followed by validation in independent GEO cohorts. **Results:** Single-cell transcriptomic analysis of 131,639 cells from 25 treatment-naïve LUAD lesions identified six CAF subtypes, including alveolar CAFs, antigen-presenting CAFs, extracellular matrix CAFs, EndMT-like CAFs, inflammatory CAFs, and myofibroblastic CAFs. CAF composition differed across pathological stages, with MIA lesions showing a distinct enrichment of eCAFs and reduced proportions of inflammatory and myofibroblastic CAF populations. Compared with pre-invasive lesions, IA lesions exhibited increased proportions of exhausted CD4+ and CD8+ T cells together with reduced cytotoxic features. Cell–cell communication analysis identified enhanced epithelial–CAF interactions in IA, including enrichment of MIF-CD74/CD44 signaling. Based on MIF-related genes, a machine learning prognostic signature was developed and validated in independent cohorts, consistently stratifying patients into distinct risk groups with significantly different survival outcomes. **Conclusions:** These findings suggest that CAF-related stromal remodeling is associated with immune suppression during LUAD progression. MIF-mediated epithelial–CAF communication may be involved in the formation of an immunosuppressive microenvironment and is associated with poor prognosis. The MIF-related signature may provide a useful approach for prognostic stratification in LUAD.

## 1. Introduction

Lung adenocarcinoma (LUAD) is the most common subtype of non-small cell lung cancer and is now more frequently detected at early stages with the widespread use of high-resolution computed tomography screening [1]. Early LUAD is generally regarded as a stepwise pathological process, progressing from atypical adenomatous hyperplasia (AAH) to adenocarcinoma in situ (AIS), minimally invasive adenocarcinoma (MIA), and ultimately invasive adenocarcinoma (IA) [2]. These lesions differ markedly in biological behavior and clinical outcome. AAH, AIS, and MIA are usually associated with a favorable prognosis after local treatment, whereas IA carries a higher risk of recurrence, metastasis, and disease-related death [3]. Defining the biological changes that accompany this transition is important for understanding malignant progression and improving risk assessment in LUAD.

Previous studies have described the genomic and transcriptional alterations that occur during LUAD evolution [4]. Multi-region sequencing and single-cell analyses have shown that malignant epithelial cells progressively acquire lineage plasticity, stem-like features, and invasive programs as lesions advance [5,6]. However, tumor progression is not determined by epithelial cells alone. The tumor microenvironment, including stromal, immune, endothelial, and extracellular matrix components, also contributes to tumor initiation, immune escape, invasion, and treatment resistance. Interactions between malignant epithelial cells and non-malignant cells may influence the shift from early lesions to invasive tumors. Although tumor microenvironmental heterogeneity has been increasingly recognized in established LUAD, how stromal and immune compartments change across the AAH-AIS-MIA-IA sequence remains insufficiently explored.

Cancer-associated fibroblasts (CAFs) are among the most abundant and functionally diverse stromal cell populations in solid tumors [7]. Rather than representing a uniform cell type, CAFs comprise multiple subpopulations with distinct phenotypes and biological functions, including extracellular matrix remodeling, cytokine secretion, angiogenesis regulation, antigen presentation, and immune modulation [8]. Depending on disease stage, CAFs may exert either tumor-restraining or tumor-promoting effects. In advanced cancers, specific CAF subsets have been implicated in immune exclusion, T-cell dysfunction, extracellular matrix deposition, and metastasis [9]. Previous studies have demonstrated substantial CAF heterogeneity within the LUAD microenvironment and identified multiple CAF subpopulations with distinct biological functions [10]. In addition, increasing evidence suggests that stromal components actively participate in the evolution of LUAD from pre-invasive lesions to invasive disease. However, how CAF populations are dynamically remodeled during this progression process and how these changes relate to immune suppression and intercellular communication remain incompletely understood.

Macrophage migration inhibitory factor (MIF) is a multifunctional cytokine involved in inflammation, tumor progression, and immune regulation [11,12]. By binding to receptors such as CD74 and CD44, MIF can activate signaling pathways related to cell survival, inflammatory responses, and immune evasion [13]. MIF signaling has been implicated in tumor–stromal and tumor–immune interactions in several malignancies. Nevertheless, whether MIF-associated communication participates in CAF remodeling and immune microenvironment evolution during LUAD progression remains unclear. Clarifying the distribution and activity of MIF-related epithelial–stromal interactions across different pathological stages may provide insights into how stromal remodeling contributes to immune suppression and invasive progression.

Single-cell RNA sequencing provides an opportunity to characterize cellular heterogeneity and intercellular communication during tumor evolution at high resolution. Although previous studies have described CAF heterogeneity in established LUAD and reported multiple CAF subtypes, how CAF populations dynamically remodel across the full pathological continuum from AAH to AIS, MIA, and IA remains incompletely understood. In addition, the relationship between CAF remodeling, immune dysfunction, and epithelial–stromal communication during LUAD progression has not been systematically investigated. We therefore performed an integrated analysis of single-cell and bulk transcriptomic datasets to characterize CAF heterogeneity, explore stage-associated changes in stromal and immune compartments, identify potential communication pathways involved in LUAD progression, and evaluate their prognostic relevance.

## 2. Methods

### 2.1. Data Collection

The scRNA-seq dataset was obtained from the Genome Sequence Archive (GSA) of the Beijing Institute of Genomics under accession number HRA001130 [14]. The scRNA-seq dataset included 25 treatment-naïve patients, comprising 3 AAH, 5 AIS, 9 MIA, and 8 IA lesions, together with matched adjacent normal tissues. The scRNA-seq data were used to assess cellular composition, CAF heterogeneity, immune cell states, inferred developmental trajectories, and cell–cell communication during LUAD progression. External transcriptomic cohorts with survival information were obtained from The Cancer Genome Atlas LUAD cohort (TCGA-LUAD) and the Gene Expression Omnibus (GEO), including GSE31210 [15] and GSE50081 [16]. To minimize platform-related variability, GEO datasets generated on the GPL570 platform with sufficient sample size and available clinical follow-up were selected. These bulk cohorts were used to develop and validate the MIF-related prognostic signature. This study was reported in accordance with the Reporting Recommendations for Tumor Marker Prognostic Studies (REMARK) guidelines [17].

### 2.2. Single-Cell RNA Sequencing Data Processing

Raw scRNA-seq data were processed using Cell Ranger software (version 3.0) in a Linux environment. The resulting barcode, feature, and matrix files were converted into a 10X-compatible format and analyzed in R (version 4.1.2). Seurat (version 4.3.0) was used for object construction, quality control, normalization, variable gene selection, dimensionality reduction, clustering, and visualization [18]. Cells were retained for downstream analysis if they contained 300–10,000 detected genes, 1000–30,000 unique molecular identifiers (UMIs), and a mitochondrial gene fraction below 15%. To minimize the influence of low-quality cells and potential doublets, stringent quality-control filtering was applied based on the number of detected genes, UMI counts, and mitochondrial gene percentages. Cells exhibiting abnormally high transcript counts suggestive of potential multiplets were excluded prior to downstream analyses. To reduce inter-sample batch effects, samples were integrated using the anchor-based integration workflow implemented in Seurat. Briefly, integration anchors were identified across samples using the FindIntegrationAnchors function, followed by batch-effect correction and data integration using the IntegrateData function. This approach aligns shared biological cell states across samples while minimizing technical variation. Following data integration, unsupervised clustering was performed and visualized using uniform manifold approximation and projection (UMAP). Major cell types were annotated based on canonical marker genes reported in previous studies. The parameters used for quality control and downstream analyses were selected according to established practices in previous single-cell LUAD studies.

### 2.3. Differential Expression Analysis

Differentially expressed genes were identified using the FindMarkers function implemented in Seurat with the Wilcoxon rank-sum test. Unless otherwise specified, genes with *p* < 0.01 were considered significantly differentially expressed.

### 2.4. Single-Cell Trajectory Analysis

Trajectory inference was performed using Monocle2 (version 2.22.0) and CytoTRACE (version 0.3.3) [19]. Gene expression matrices and cell annotation information were used to construct Monocle objects. After size-factor estimation, genes with a significance threshold of *p* < 0.01 were selected for cell ordering. Dimensionality reduction was performed using the DDRTree method implemented in the reduceDimension function. CytoTRACE was used to estimate the relative differentiation status of CAF subsets and to assist in the interpretation of pseudotime trajectories.

### 2.5. SCENIC Analysis

Transcription factor activity was assessed using SCENIC (version 1.2.4) and pySCENIC (version 0.11.2) [20]. Co-expression networks were inferred using the GRNBoost2 algorithm in pySCENIC, followed by identification of candidate transcription factor target genes. Motif enrichment analysis was performed with RcisTarget using the 500bp-upstream-7species.mc9nr database from the cisTarget resource. Regulon activity was quantified by area under the curve scores, and regulons activated in more than 20% of cells in at least one cluster were retained for downstream analysis.

### 2.6. Assessment of Gene Enrichment Scores

AUCell (version 1.16.0) was used to estimate cell-level enrichment of functional gene sets [21,22,23,24]. Gene sets related to cytotoxicity, exhaustion, proliferation, naiveness, regulatory T-cell features, angiogenesis, and epithelial–mesenchymal transition are listed in Table S1. Enrichment scores were visualized using violin plots and compared across cell subtypes and pathological states.

### 2.7. Cell–Cell Communication Analysis

Cell–cell communication analysis was conducted using CellPhoneDB (version 3.0.0) [25] and Cellchat (version 1.6.0) [26]. Ligand–receptor interactions were predicted based on cell-type-specific expression profiles. Interaction strength and communication probability were summarized across pathological states and cell populations. The analysis focused on epithelial–CAF interactions and MIF-related ligand–receptor pairs, particularly MIF–CD74/CD44 signaling. Heatmaps, dot plots, and network plots were used to display the predicted communication patterns.

### 2.8. Functional Enrichment Analysis

Functional enrichment analyses were conducted using clusterProfiler (Version 4.7.1). Differentially expressed genes were analyzed for Gene Ontology (GO) and Kyoto Encyclopedia of Genes and Genomes (KEGG) pathway analyses [27]. Enrichment results were visualized with ggplot2 (version 3.4.1), and *p* < 0.05 was considered statistically significant. Gene Set Variation Analysis (GSVA) was used to estimate pathway activity based on hallmark gene sets [28].

### 2.9. Machine Learning Model

A machine learning-based framework was used to construct an MIF-related prognostic signature [29]. Candidate MIF-related genes were identified from an MIF-centered protein–protein interaction network constructed following cell–cell communication analysis, which highlighted MIF signaling as a key pathway associated with LUAD progression.

Ten commonly used machine learning algorithms were considered, including CoxBoost, elastic net, generalized boosted regression modeling, Lasso, partial least squares regression for Cox, random survival forest, Ridge, stepwise Cox, supervised principal components, and survival support vector machine. In total, 116 algorithmic combinations were initially examined in the TCGA-LUAD cohort. To reduce overfitting and model selection bias, model development was restricted to the training cohort and conducted under a leave-one-out cross-validation framework. For each candidate model, supervised feature selection, parameter tuning, and coefficient estimation, when applicable, were performed within the corresponding resampling procedure of the training data. The left-out sample was not used during feature selection or model fitting, thereby preserving the separation between model training and internal performance estimation. Because the number of candidate MIF-related genes was limited, some algorithmic combinations were not applicable or did not generate stable models under the predefined modeling procedure. Therefore, 66 eligible algorithmic combinations were retained for subsequent assessment. Candidate models were compared based on their internal cross-validation performance, model stability, and parsimony within the TCGA-LUAD training cohort. After the final model structure was determined, the model was refitted in the entire training cohort, and the risk-score formula and coefficients were fixed. The independent GEO cohorts were used only for external validation of the locked model and were not involved in feature selection, hyperparameter optimization, coefficient estimation, or iterative model refinement. Model performance was evaluated using Harrell’s concordance index in each independent validation cohort, and the average validation C-index was used to summarize cross-cohort generalizability. Patients were assigned to high- and low-risk groups according to the optimal cutoff determined exclusively in the training cohort. Prognostic performance was further assessed using Kaplan–Meier survival curves and time-dependent receiver operating characteristic curves.

### 2.10. Model Generalizability Evaluation

To evaluate the generalizability of different models, we collected previously published lung cancer-related signatures developed for prognosis prediction, risk stratification, molecular classification, or treatment-response assessment and compared them with our model across the validation cohorts. The collected signatures represented a broad spectrum of lung cancer biology, encompassing tumor immune and inflammatory regulation; cell-state programs such as stemness and epithelial–mesenchymal transition; stress- and death-related processes, including autophagy and ferroptosis; metabolic and epigenetic alterations; oncogenic signaling pathways; aging-related features; and treatment-response phenotypes. For each study, the signature score was recalculated based on the scoring method reported in the original study. The C-index was then calculated for each signature to compare their predictive performance and cross-cohort stability.

### 2.11. Survival Analysis

Survival analyses were conducted using the survival and survminer R packages. Kaplan–Meier curves were generated to compare overall survival between high- and low-risk groups, and differences were assessed using the log-rank test. Cox proportional hazards models were used to estimate hazard ratios and corresponding confidence intervals.

### 2.12. In Vitro Validation of MIF-Associated Fibroblast Activation

The human LUAD cell line A549 was obtained from the Cell Bank of the Chinese Academy of Sciences (Shanghai, China). Human lung fibroblast (HFL1) was purchased from the American Type Culture Collection (ATCC, Manassas, VA, USA). All cell lines were authenticated by short tandem repeat profiling and routinely tested to confirm the absence of mycoplasma contamination. To provide experimental support for the computationally inferred MIF-CD74/CD44 signaling axis, conditioned medium (CM) experiments were performed using LUAD cells and lung fibroblasts. A549 cells were cultured in RPMI 1640 medium containing 10% fetal bovine serum (FBS). Upon reaching approximately 80% confluence, cells were washed with phosphate-buffered saline and incubated in serum-reduced medium (1640 containing 1% FBS) for 24 h. The culture supernatant was collected, centrifuged at 300× *g* for 5 min to remove cellular debris, and filtered through a 0.22 μm filter to obtain LUAD-conditioned medium (LUAD-CM). HFL1 cells were cultured in Ham’s F-12K medium containing10% FBS. Fibroblasts were divided into three groups: (1) fibroblast control, cultured in fresh medium; (2) LUAD-CM, cultured in a 1:1 mixture of LUAD-conditioned medium and fresh medium; and (3) LUAD-CM plus anti-MIF antibody, cultured in LUAD-conditioned medium supplemented with a neutralizing anti-MIF antibody (10 μg/mL). Cells were incubated for 48 h before analysis. For flow cytometric analysis, fibroblasts were harvested and stained with fluorochrome-conjugated antibodies against CD74 and CD44. Mean fluorescence intensity (MFI) and the proportion of positive cells were quantified using FlowJo software(version 10.8.1).

### 2.13. Quantitative Real-Time PCR

Total RNA was extracted from fibroblasts using TRIzol Reagent (Invitrogen, Thermo Fisher Scientific, Waltham, MA, USA) according to the manufacturer’s instructions. RNA concentration and purity were determined using a NanoDrop 2000 spectrophotometer (Thermo Fisher Scientific, USA). Complementary DNA (cDNA) was synthesized from 1 μg of total RNA using the PrimeScript RT Reagent Kit (Takara Bio, Shiga, Japan). Quantitative real-time PCR (qRT-PCR) was performed using TB Green Premix Ex Taq II (Takara Bio, Japan) on an ABI QuantStudio 5 Real-Time PCR System (Applied Biosystems, Foster City, CA, USA). The PCR cycling conditions were as follows: initial denaturation at 95 °C for 30 s, followed by 40 cycles of 95 °C for 5 s and 60 °C for 30 s. Melt curve analysis was performed to verify amplification specificity. FAP mRNA expression was normalized to ACTB (β-actin), and relative gene expression levels were calculated using the 2^−ΔΔCt^ method. All reactions were performed in triplicate.

The primer sequences used for qRT-PCR were as follows:

FAP:

Forward, 5′-TGAACGAGTATGTTTGCAGTGG-3′;

Reverse, 5′-GGTCTTTGGACAATCCCATGT-3′.

ACTB:

Forward, 5′-CATGTACGTTGCTATCCAGGC-3′;

Reverse, 5′-CTCCTTAATGTCACGCACGAT-3′.

## 3. Results

### 3.1. Single-Cell Transcriptional Atlas Reveals Stromal and Immune Remodeling During LUAD Progression

**Figure 1:**
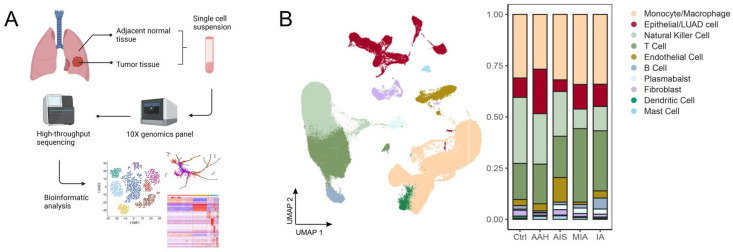
Overview of single-cell transcriptomic profiling of LUAD lesions and matched adjacent normal tissues: (**A**) Schematic workflow illustrating data processing and single-cell transcriptomic analysis of the tumor microenvironment in LUAD. (**B**) UMAP visualization of all cells profiled by single-cell RNA sequencing, colored according to annotated major cell types.

The study workflow is shown in Figure 1A. To examine microenvironmental changes during LUAD progression, we analyzed a public scRNA-seq dataset comprising 25 treatment-naïve patients, including 3 AAH, 5 AIS, 9 MIA, and 8 IA cases, together with matched adjacent normal tissues [14]. After quality control, 131,639 single-cell transcriptomes were retained from lesion tissues and matched adjacent normal tissues. Among lesion-derived cells, 3222 were from AAH, 16,340 from AIS, 34,278 from MIA, and 24,997 from IA. Quality-control metrics, including normalized UMIs, numbers of detected genes, and mitochondrial gene fractions, are shown in Figure S1A.

Unsupervised clustering identified 33 cell clusters in the LUAD microenvironment (Figure S1B). These clusters were annotated into major cell populations based on canonical marker genes, including T cells, B cells, plasma cells, fibroblasts, monocytes/macrophages, natural killer cells, dendritic cells, mast cells, epithelial/LUAD cells, and endothelial cells (Figure 1B and Figure S1C). The relative abundance of stromal and immune cell populations differed across pathological stages (Figure S1D). Fibroblast and endothelial cell fractions showed notable variation among groups, suggesting that non-epithelial compartments may be involved in LUAD progression. We therefore focused subsequent analyses on CAF heterogeneity and its relationship with immune remodeling.

### 3.2. CAF Subtypes Exhibit Stage-Associated Changes in Composition and Function

**Figure 2:**
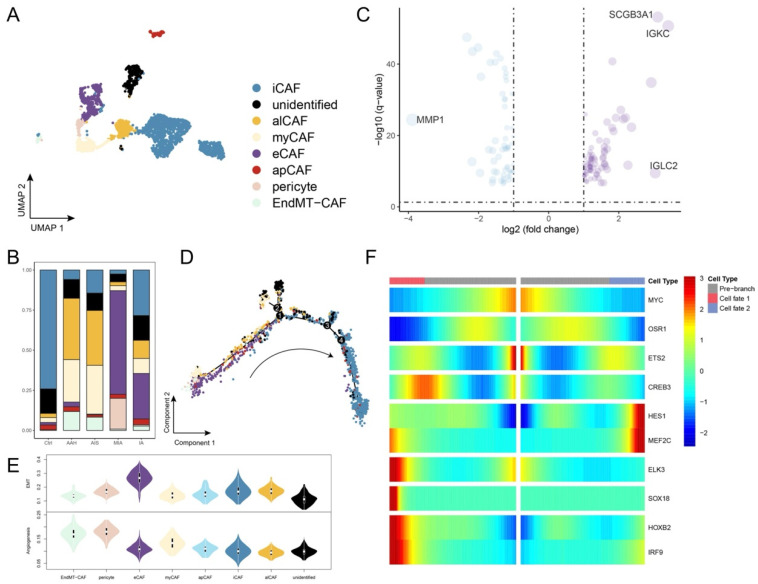
Characterization of CAF heterogeneity across pathological states of LUAD. (**A**) UMAP visualization of cancer-associated fibroblasts (CAFs) from AAH, AIS, MIA, IA, and adjacent normal tissues, colored by CAF subtype. (**B**) Bar plot showing the relative proportions of each CAF subtype across the four pathological states and adjacent normal tissues. (**C**) Volcano plot showing differentially expressed genes in extracellular matrix CAFs between MIA and IA lesions. (**D**) Pseudotime trajectory analysis of CAFs across the progression from AAH to IA. (**E**) Violin plots showing functional signature scores across CAF subtypes. (**F**) Heatmaps showing the expression dynamics of representative marker genes along the inferred CAF differentiation trajectory.

CAFs are involved in several tumor-related processes, including extracellular matrix remodeling, cytokine production, immune regulation, angiogenesis, and invasion [30]. To examine CAF heterogeneity during LUAD progression, fibroblast-lineage cells were extracted and reclustered. Six CAF subsets were identified based on established marker genes and functional features: alveolar CAFs (alCAFs), antigen-presenting CAFs (apCAFs), extracellular matrix CAFs (eCAFs), EndMT-like CAFs, inflammatory CAFs (iCAFs), and myofibroblastic CAFs (myCAFs) (Figure 2A and Figure S2A). These subsets were generally consistent with CAF populations reported in previous single-cell studies [31].

The distribution of CAF subsets varied across pathological stages (Figure 2B) [10]. MIA samples showed a CAF composition distinct from the other tumor states, with lower proportions of iCAFs, myCAFs, EndMT-like CAFs, and alCAFs and a relatively higher proportion of eCAFs. We next compared the transcriptional profiles of eCAFs from MIA and IA lesions. Differential expression analysis identified 142 genes with stage-associated expression differences (*p* < 0.01). SCGB3A1 was more highly expressed in MIA-derived eCAFs, whereas IA-derived eCAFs showed increased expression of genes related to immune regulation and cell differentiation (Figure 2C and Figure S2B). These results suggest that eCAFs acquire altered functional features during the transition from minimally invasive to invasive disease.

Trajectory analysis was then used to explore potential CAF-state transitions. Pseudotime ordering suggested a continuum from EndMT-like CAF and pericyte-associated states toward iCAF-enriched states, with myCAFs and apCAFs located in intermediate regions (Figure 2D). CytoTRACE analysis further supported lower differentiation scores in EndMT-like CAF and pericyte-like populations and higher differentiation scores in inflammatory or alveolar CAF states (Figure S2C). Functional scoring showed that EndMT-like CAFs had the highest angiogenesis-related scores, whereas iCAFs showed relatively low angiogenesis scores (Figure 2E). Genes associated with tumor-promoting programs tended to increase along the inferred CAF trajectory (Figure 2F). These findings indicate that CAFs undergo stage-related compositional and functional changes during LUAD progression.

### 3.3. Transcriptional Regulatory Programs Differ Across CAF States

To investigate regulatory programs associated with CAF heterogeneity, we used SCENIC to infer transcription factor activity across CAF subsets. Distinct regulon activation patterns were observed among CAF states (Figure S2D). HOXB2 and IRF9 regulons [32,33] were activated in selected CAF populations. Regulons previously associated with tumorigenesis, invasion, or metastasis, including ETS2 [34], HES1 [35], ELK3 [36], SOX18 [37], and MYC [38], also showed differential enrichment across EndMT-like CAFs, iCAFs, myCAFs, and eCAFs. KEGG analysis of EndMT-like CAFs further showed enrichment of pathways related to cell migration and stromal remodeling (Figure S2E). These results suggest that CAF states are associated with distinct transcriptional regulatory programs, which may underlie their functional differences during LUAD progression.

### 3.4. T-Cell Dysfunction Accompanies Stromal Remodeling in Invasive LUAD

**Figure 3:**
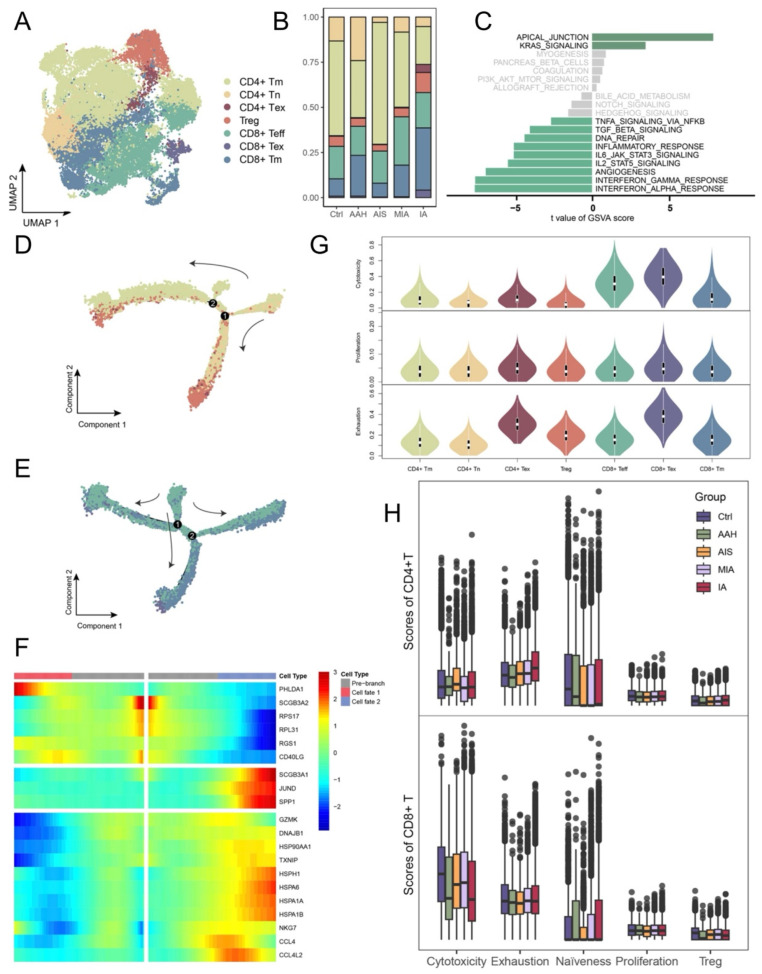
Dynamic remodeling of CD4^+^ and CD8^+^ T-cell states during LUAD progression. (**A**) UMAP visualization of CD4^+^ and CD8^+^ T cells from AAH, AIS, MIA, IA, and adjacent normal tissues, colored by T-cell subtype. (**B**) Relative proportions of T-cell clusters across pathological states. (**C**) Differences in pathway activities of CD8^+^ T cells between MIA and IA lesions, as assessed by GSVA at the single-cell level. The lower and upper panels represent MIA- and IA-derived CD8^+^ T cells, respectively, with t values estimated using a linear model. Black arrows indicate pathways or genes highlighted in the main text. (**D**) Pseudotime trajectory analysis of CD4^+^ naïve T cells, CD4^+^ memory T cells, CD4^+^ exhausted T cells, and regulatory T cells. (**E**) Pseudotime trajectory analysis of CD8^+^ memory T cells, CD8^+^ effector T cells, and CD8^+^ exhausted T cells. (**F**) Heatmap showing dynamic changes in marker gene expression along the inferred T-cell developmental trajectories, identified by BEAM analysis. (**G**) Functional signature scores for cytotoxicity, proliferation, and exhaustion across T-cell clusters. (**H**) Functional signature scores for cytotoxicity, exhaustion, naiveness, proliferation, and Treg-related programs in CD4^+^ and CD8^+^ T cells across pathological states.

Given the role of CAFs in modulating anti-tumor immunity [39], we next examined T-cell states across LUAD progression. T cells were reclustered into major functional subsets, including CD4^+^ memory T cells, CD4^+^ naïve T cells, CD4^+^ exhausted T cells, regulatory T cells, CD8^+^ memory T cells, CD8^+^ effector T cells, and CD8^+^ exhausted T cells (Figure 3A). Compared with earlier pathological stages, IA samples contained higher proportions of CD4^+^ exhausted T cells, regulatory T cells, and CD8^+^ exhausted T cells (Figure 3B), indicating a more dysfunctional T-cell compartment in invasive lesions.

Pseudotime analysis of CD8 T cells suggested a shift from effector-like states toward exhausted states, accompanied by reduced expression of cytotoxicity-associated genes, including GZMK and NKG7 (Figure 3C–F). Functional scoring was consistent with these changes. Although CD8 effector and exhausted T cells showed relatively high cytotoxicity scores overall, IA-derived CD8 T cells exhibited lower cytotoxic features than MIA-derived CD8 T cells, whereas exhaustion and proliferation scores increased with tumor progression (Figure 3G,H). These findings indicate that the transition to invasive LUAD is accompanied by T-cell dysfunction, providing immune-contextual evidence for the development of an immunosuppressive microenvironment alongside CAF remodeling.

### 3.5. MIF-Associated Epithelial–CAF Communication Is Increased in Invasive Lesions

**Figure 4:**
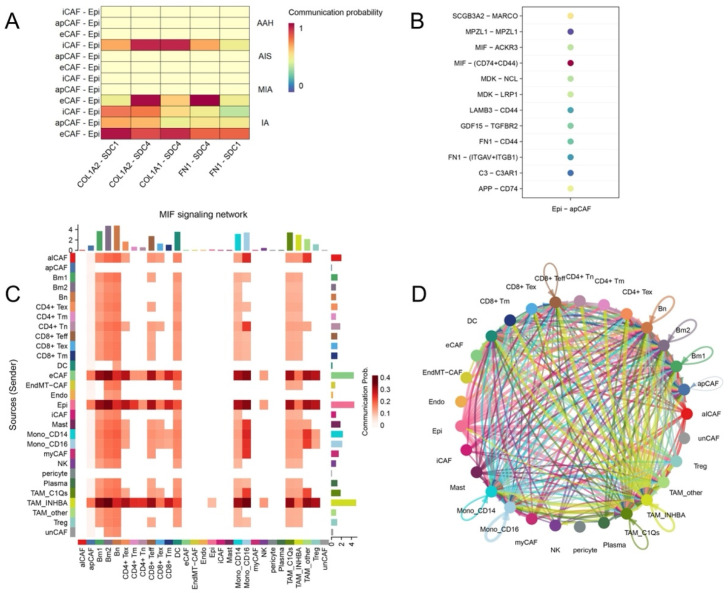
Cell–cell communication analysis of the LUAD tumor microenvironment: (**A**) Interaction strength between epithelial cells and major CAF subtypes, including iCAFs, apCAFs, and eCAFs, across pathological states. (**B**) Dot plot showing the predicted communication probability of ligand–receptor interactions between epithelial cells and apCAFs in IA lesions. (**C**) Heatmap showing inferred MIF signaling activity among cell populations in IA lesions. (**D**) Network visualization of MIF-mediated cell–cell communication in IA lesions.

To examine intercellular communication across pathological stages, ligand–receptor analyses were performed using CellPhoneDB and CellChat. CAF subsets, particularly apCAFs, eCAFs, and iCAFs, were frequently involved in predicted communication networks (Figure S3A–E). Given the stage-associated changes in CAF composition, we further examined interactions between epithelial cells and CAF subsets across early and invasive lesions. In IA, epithelial cells showed increased predicted interactions with apCAFs, eCAFs, and iCAFs. Several extracellular matrix- and metastasis-related ligand–receptor pairs, including COL1A-SDC and FN1-SDC, were enriched between CAF subsets and epithelial cells (Figure 4A). Epithelial–apCAF communication was most evident in IA and was less apparent in AAH, AIS, and MIA. In addition, communication between EndMT-like CAFs and apCAFs increased with disease progression, suggesting greater complexity of CAF-CAF and epithelial–CAF interaction networks in invasive lesions.

Among the predicted pathways, MIF-related signaling was notable. MIF-CD74/CD44 interactions contributed to epithelial–CAF communication, particularly between epithelial cells and apCAFs in IA (Figure 4B). CellChat analysis also showed MIF signaling activity across pathological stages, with greater involvement of CAF subsets in IA (Figure 4C,D and Figure S4A–F). Given the reported roles of MIF in inflammation, tumor progression, and immune regulation, these results suggest that MIF-associated epithelial–CAF communication may be related to immune suppression and invasive progression in LUAD. These findings are based on transcriptomic prediction of ligand–receptor interactions and should be validated in functional studies.

### 3.6. LUAD-Conditioned Medium Induces CD74/CD44 Expression and Fibroblast Activation

To experimentally evaluate the predicted MIF-CD74/CD44 epithelial–CAF communication axis, lung fibroblasts were exposed to conditioned medium derived from LUAD cells. Flow cytometric analysis demonstrated that treatment with LUAD-conditioned medium significantly increased the expression of CD74 and CD44 in fibroblasts compared with control fibroblasts. Neutralization of MIF partially attenuated the upregulation of both receptors, suggesting that tumor-derived MIF contributes to the induction of an MIF-responsive fibroblast phenotype (Figure S5A–C, Table S2). Consistent with these findings, qRT-PCR analysis revealed increased expression of the CAF-activated marker FAP following LUAD-conditioned medium stimulation. The induction of FAP was partially reversed by MIF blockade. These results indicate that soluble factors released by LUAD cells, including MIF, may promote fibroblast activation and enhance the expression of MIF-associated receptor components. Taken together, these data provide experimental support for the computationally predicted MIF-CD74/CD44 signaling axis and suggest that MIF-mediated epithelial–fibroblast communication may contribute to stromal remodeling during LUAD progression.

### 3.7. An MIF-Related Prognostic Signature Stratifies Survival Risk in LUAD

**Figure 5:**
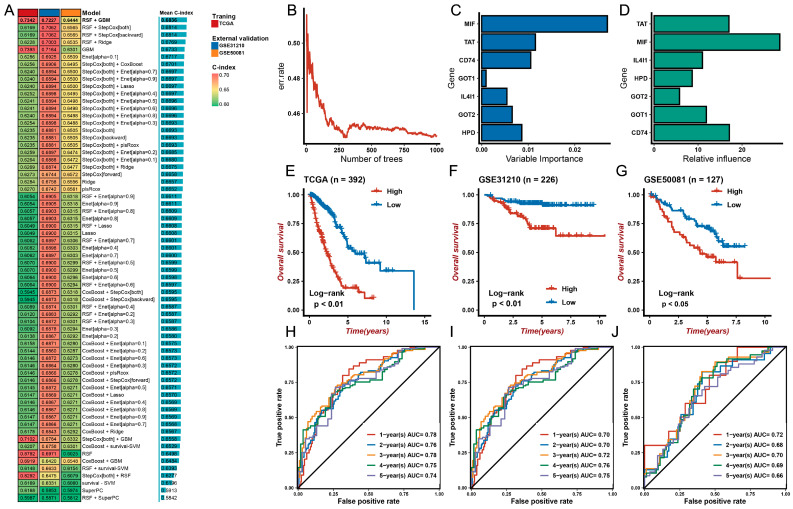
Development and validation of a machine learning-based MIF-related prognostic signature: (**A**) Overview of the integrative machine learning procedure. A total of 116 prediction models were constructed using a leave-one-out cross-validation framework, and the C-index of each model was evaluated across validation cohorts. (**B**–**D**) Dimension reduction and prognostic gene selection based on the random survival forest algorithm, identifying seven prognostic genes for model construction. (**E**–**G**) Kaplan–Meier survival curves comparing overall survival between high- and low-risk groups stratified by the MIF-related signature score in the TCGA-LUAD, GSE31210, and GSE50081 cohorts. (**H**–**J**) Time-dependent ROC curves evaluating the predictive performance of the MIF-related signature for 1-, 2-, 3-, 4-, and 5-year overall survival in the TCGA-LUAD, GSE31210, and GSE50081 cohorts.

To assess the clinical relevance of the MIF-associated communication identified in the single-cell analyses, an MIF-centered protein–protein interaction network was constructed (Figure S6). Candidate genes derived from this network were subsequently used for prognostic model development. In the TCGA-LUAD cohort, 116 algorithm combinations were initially tested using a leave-one-out cross-validation framework, and 66 models were retained for final evaluation. The best-performing exploratory model combined random survival forest and generalized boosted regression modeling, with a concordance index of 0.684 (Figure 5A–D). This model showed stable performance across the validation datasets and compared favorably with other algorithm combinations and published signatures (Figure S7).

Patients were stratified into high- and low-risk groups according to the MIF-related signature. High-risk patients had significantly shorter overall survival in TCGA-LUAD, GSE31210, and GSE50081 (Figure 5E–G). Time-dependent receiver operating characteristic analysis further supported the prognostic performance of the signature. The 1-, 2-, 3-, 4-, and 5-year AUC values were 0.78, 0.76, 0.78, 0.75, and 0.74 in TCGA-LUAD; 0.70, 0.70, 0.72, 0.76, and 0.75 in GSE31210; and 0.72, 0.68, 0.70, 0.69, and 0.66 in GSE50081, respectively (Figure 5H–J). These results indicate that the MIF-related signature is associated with survival outcomes in LUAD and may reflect stromal and immune features linked to poor prognosis.

## 4. Discussion

In this study, we examined stromal and immune changes during the stepwise progression of LUAD from AAH, AIS, and MIA to IA. By integrating single-cell transcriptomic analysis with external bulk cohorts, we identified heterogeneous CAF populations and observed stage-related differences in CAF composition, functional features, and predicted cell–cell communication. Invasive lesions showed increased epithelial–CAF interactions, enhanced MIF-related signaling, and a more immunosuppressive immune context marked by T-cell dysfunction. We also developed an MIF-related prognostic signature that stratified patients into different risk groups across independent cohorts. These findings suggest that CAF remodeling is linked to immune suppression during LUAD progression and may have clinical relevance.

Previous studies have characterized the evolutionary landscape of LUAD progression and demonstrated progressive changes in epithelial cell states, genomic alterations, and metabolic programs during the transition from preinvasive lesions to invasive adenocarcinoma [14]. More recently, single-cell analyses have revealed substantial heterogeneity within the tumor microenvironment, including diverse stromal and immune cell populations [10]. However, the dynamic changes of CAF populations across the AAH-AIS-MIA-IA sequence remain incompletely understood. In the present study, we observed marked differences in CAF composition across pathological stages. MIA lesions displayed a relatively higher proportion of eCAFs and lower proportions of iCAFs, EndMT-like CAFs, and myCAFs, whereas invasive lesions showed enrichment of CAF populations associated with inflammatory signaling and intercellular communication. These observations suggest that CAF composition changes during LUAD progression and may reflect alterations in tumor–stromal interactions accompanying disease evolution.

CAFs are increasingly recognized as a heterogeneous stromal population with diverse biological functions [30,39]. Depending on the tumor context, CAFs may participate in extracellular matrix remodeling, angiogenesis, immune regulation, and tumor invasion. Consistent with previous studies, we identified multiple CAF subpopulations with distinct transcriptional and functional characteristics. Trajectory analysis suggested potential transitions among CAF states, and functional analyses revealed differences in angiogenesis-related and tumor-associated programs across CAF subsets. Although the biological roles of individual CAF populations require further investigation, the enrichment of inflammatory and communication-associated CAF states in invasive lesions is consistent with a progressively remodeled stromal microenvironment during disease progression.

Among the predicted ligand–receptor interactions, MIF signaling was one of the most prominent pathways identified in invasive lesions. MIF is a multifunctional cytokine involved in inflammation, tumor progression, and immune regulation [40]. We observed increased MIF-CD74/CD44-mediated communication between epithelial cells and CAF subsets, particularly apCAFs, in IA lesions. Previous studies have shown that MIF signaling contributes to tumor-promoting inflammation and immune evasion in several cancer types. Our findings suggest that MIF-associated epithelial–CAF communication may become increasingly involved during LUAD progression. However, because ligand–receptor analyses are based on transcriptomic inference, these observations should be interpreted as evidence of potential communication rather than direct proof of active signaling. Notably, several genes retained in the final signature, including TAT, GOT1, GOT2, and HPD, are involved in amino acid metabolism. Although these genes are not canonical components of MIF signaling, they were identified through an MIF-centered protein–protein interaction network and subsequent machine learning selection. Emerging evidence indicates that MIF contributes to metabolic reprogramming, mitochondrial adaptation, and immunometabolic regulation within the tumor microenvironment [41,42]. Therefore, the final signature may capture both direct MIF signaling and downstream metabolic consequences associated with MIF-mediated stromal–immune interactions. This observation further supports the concept that MIF functions as a broader regulator of tumor microenvironmental adaptation rather than solely as an inflammatory cytokine.

To provide preliminary experimental support for the computationally inferred MIF-CD74/CD44 signaling axis, we performed an in vitro conditioned-medium assay using LUAD cells and lung fibroblasts. Exposure of fibroblasts to tumor-derived conditioned medium induced the expression of CD74, CD44, and the CAF-associated marker FAP, whereas MIF neutralization partially attenuated these effects. Although this simplified in vitro system does not fully recapitulate the complexity of the tumor microenvironment, the findings are consistent with the predicted epithelial–CAF communication network identified by CellPhoneDB and CellChat analyses. These results suggest that tumor-derived MIF may participate in fibroblast activation and receptor remodeling, providing independent support for the biological relevance of the MIF-CD74/CD44 axis. Future studies incorporating patient tissues, multiplex immunofluorescence, spatial transcriptomics, and functional perturbation experiments will be necessary to further define the spatial organization and mechanistic significance of MIF-mediated stromal interactions in LUAD.

The immune compartment also changed substantially across pathological stages. Compared with earlier lesions, IA samples contained higher proportions of exhausted CD4^+^ and CD8^+^ T cells and exhibited reduced cytotoxic features. These findings are consistent with progressive impairment of anti-tumor immune activity during LUAD development. The coexistence of enhanced epithelial–CAF communication and T-cell dysfunction raises the possibility that stromal remodeling contributes to the establishment of an immunosuppressive microenvironment [43]. Although the present study does not establish a direct mechanistic relationship, MIF-associated signaling may represent one pathway linking CAF activity to immune dysfunction. Future studies incorporating spatial transcriptomics, multiplex imaging, and functional co-culture systems will be needed to determine whether MIF-enriched CAF niches directly influence T-cell infiltration, activation, or effector function.

An important question is whether the observed CAF changes actively contribute to disease progression or simply reflect changes that occur during tumor evolution. Because the present study analyzed cross-sectional samples collected from different patients at different pathological stages, the observed differences cannot be interpreted as direct evidence of temporal progression. Inter-patient heterogeneity, sampling bias, and local microenvironmental variation may also contribute to the observed CAF distributions. Therefore, our findings should be interpreted as evidence of stage-associated CAF remodeling rather than proof that specific CAF populations drive progression. Longitudinal sampling and functional studies will be required to determine whether particular CAF states actively promote the transition from preinvasive lesions to invasive LUAD.

To further evaluate the potential clinical relevance of these findings, we developed an MIF-related prognostic signature based on genes derived from an MIF-centered protein–protein interaction network that was generated following the communication analyses. Unlike many previously reported prognostic models that are derived directly from bulk transcriptomic data, the candidate genes in our model were selected based on biological observations from single-cell analyses. The signature consistently stratified patients into high- and low-risk groups across TCGA-LUAD and two independent GEO cohorts, with higher risk scores associated with poorer survival outcomes [7,15]. These findings suggest that the signature may capture stromal and immune features associated with disease aggressiveness. Nevertheless, the model remains exploratory and requires further validation in prospective cohorts with detailed clinical and molecular information.

The findings of this study may have several potential clinical implications. First, MIF-associated CAF remodeling may help identify patients with a biologically aggressive tumor microenvironment, including some tumors that are classified as early stage by conventional pathological criteria. If validated, stromal features associated with CAF remodeling may complement existing clinicopathological factors for postoperative risk assessment. Second, MIF-associated epithelial–CAF communication may represent a candidate therapeutic target for future investigation. However, CAF populations are functionally heterogeneous, and non-selective targeting of CAFs may affect both tumor-promoting and tumor-restraining populations. Future studies should therefore focus on specific CAF states or signaling pathways rather than broad depletion of stromal cells.

Several limitations should be acknowledged. First, this study relied primarily on publicly available transcriptomic datasets and is therefore subject to the limitations of retrospective analyses, including selection bias, batch effects, and incomplete clinical annotation. Second, the number of samples available for certain pathological stages, particularly AAH and AIS, was limited. Third, pseudotime analyses infer potential developmental relationships but cannot directly reconstruct true cellular lineage trajectories. Fourth, ligand–receptor analyses predict potential communication events based on gene expression and do not confirm active signaling. Finally, although the prognostic signature was validated in external cohorts, its clinical utility requires prospective validation and comparison with established clinicopathological predictors.

## 5. Conclusions

This study describes CAF and immune remodeling during the pathological progression of LUAD. CAF subsets showed stage-associated changes in composition and function, and invasive lesions exhibited increased epithelial–CAF communication, partly involving MIF-CD74/CD44 signaling. T-cell dysfunction was also more evident in invasive disease, supporting the presence of an immunosuppressive tumor microenvironment. A machine learning-based MIF-related signature stratified patients with LUAD by survival risk across independent cohorts. These findings suggest that MIF-associated CAF remodeling is related to LUAD progression and prognosis, but further experimental and clinical validation is needed.

## Data Availability

The scRNA-seq data utilized in this study were obtained from the GSA at the Beijing Institute of Genomics, with the corresponding accession number HRA001130 (https://ngdc.cncb.ac.cn/gsa-human/browse/HRA001130, accessed on 1 November 2022). Independent survival datasets were collected from well-established repositories, including TCGA and GEO (GSE189487, GSE31210, and GSE50081).

## Supplementary Materials

The following supporting information can be downloaded at: https://www.mdpi.com/article/10.3390/biomedicines14071581/s1.

## Abbreviations

Abbreviation	Full Name
LUAD	Lung adenocarcinoma
AAH	Atypical adenomatous hyperplasia
AIS	Adenocarcinoma in situ
MIA	Minimally invasive adenocarcinoma
IA	Invasive adenocarcinoma
CAFs	Cancer-associated fibroblasts
TME	Tumor microenvironment
MIF	Macrophage migration inhibitory factor
CT	Computed tomography
scRNA-seq	Single-cell RNA sequencing
TCGA	The Cancer Genome Atlas
GEO	Gene Expression Omnibus
UMAP	Uniform manifold approximation and projection
TFs	Transcription factors
GO	Gene Ontology
KEGG	Kyoto Encyclopedia of Genes and Genomes
GSVA	Gene Set Variation Analysis
ATCC	American Type Culture Collection
CM	Conditioned medium
MFI	Mean fluorescence intensity
Enet	Elastic net
GBM	Generalized boosted regression modeling
plsRcox	Partial least squares regression for Cox
RSF	Random survival forest
SuperPC	Supervised principal components
SVM	Support vector machine
LOOCV	Leave-one-out cross-validation
ROC	Receiver operating characteristic
AUC	Area under the curve
