# Supplementary material for: MIF-Associated Immunosuppressive CAF Remodeling Predicts Poor Prognosis During Lung Adenocarcinoma Progression: A Single-Cell and Multicohort Transcriptomic Study
Source: Biomedicines. 2026 Jul 15;14(7):1581. doi: 10.3390/biomedicines14071581 (PMC13405759; doi:10.3390/biomedicines14071581)
Supplement: Supplementary file 1 [file biomedicines-14-01581-s001.zip › Figures S1-S7.pdf]

Supplementary Figure Legends

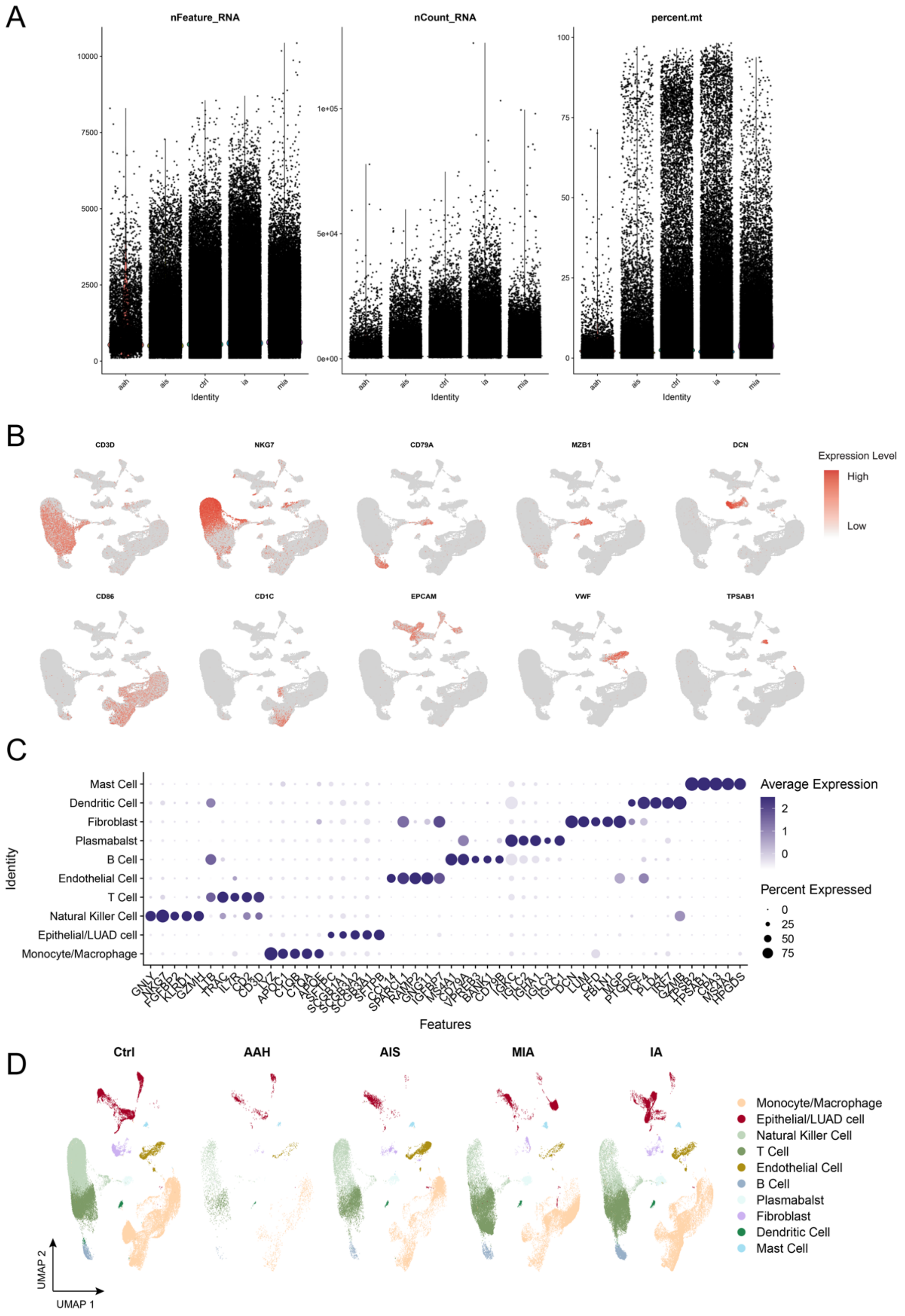

Figure S1. Quality control and annotation of major cell types in the single-cell RNA-seq dataset.

(A) Violin plots showing quality-control metrics for cells derived from the four pathological states and adjacent normal tissues.

(B) Expression patterns of canonical marker genes used to annotate major cell clusters in lung tissues.

(C) Dot plot showing differentially expressed marker genes across the annotated major cell clusters.

(D) UMAP visualization of major cell clusters stratified by tissue type.

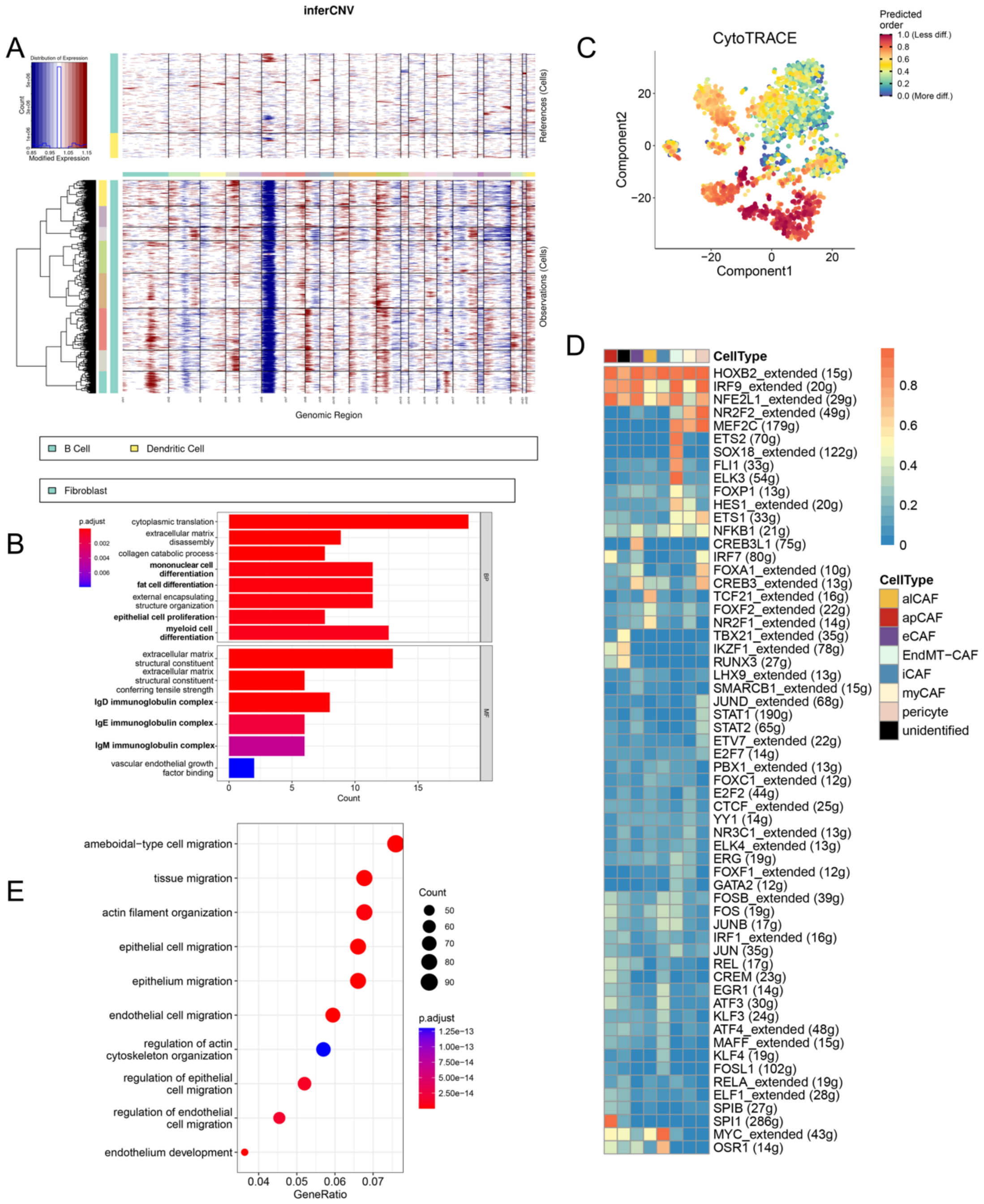

Figure S2. Additional characterization of CAF heterogeneity.

(A) Inferred copy-number variation profiles based on single-cell RNA-seq data.

(B) GO enrichment analysis of differentially expressed genes in IA-derived eCAFs and MIA-derived eCAFs. The upper and lower panels show enrichment results for IA- and MIA-derived eCAFs, respectively.

(C) CytoTRACE analysis of CAFs, colored by inferred differentiation state.

(D) Heatmap showing transcription factor regulon activity across CAF clusters.

(E) KEGG pathway enrichment analysis of EndMT-like CAFs.

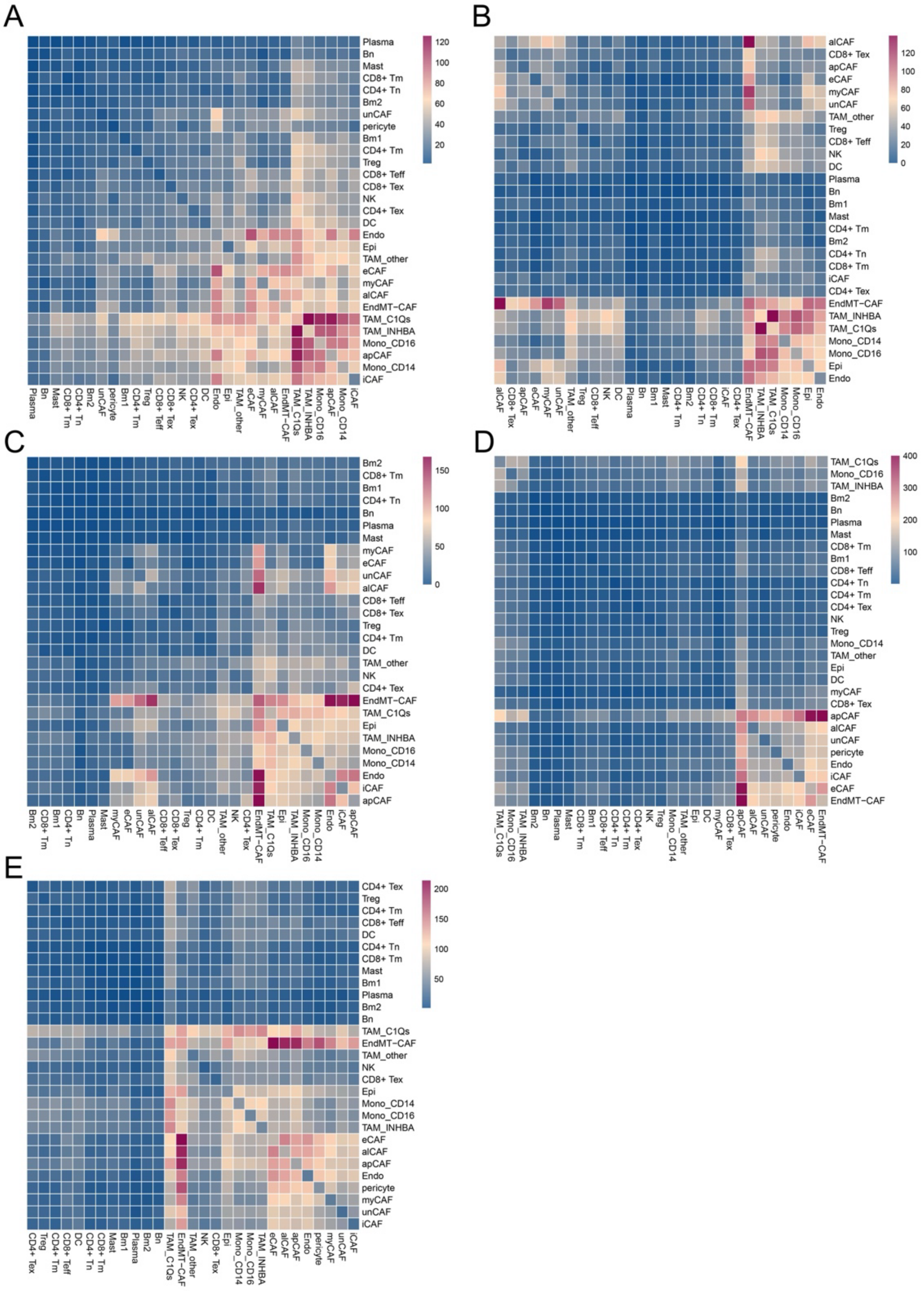

Figure S3. Global cell–cell interaction landscape across LUAD pathological states. Predicted interactions among 29 annotated cell types in (A) adjacent normal tissues, (B) AAH, (C) AIS, (D) MIA, and (E) IA lesions. The scale indicates the inferred number or strength of interactions between cell populations, with higher values representing stronger predicted communication.

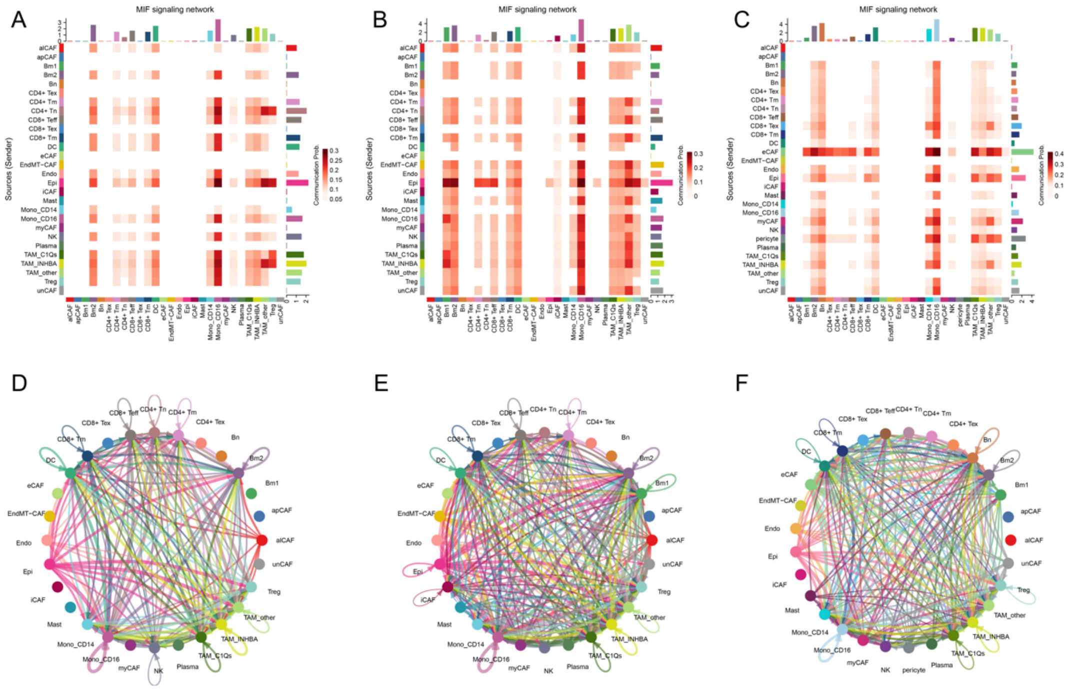

Figure S4. MIF signaling networks across early LUAD pathological states. Heatmaps showing inferred MIF signaling activity in (A) AAH, (B) AIS, and (C) MIA lesions. Network plots showing MIF-mediated cell–cell communication in (D) AAH, (E) AIS, and (F) MIA lesions.

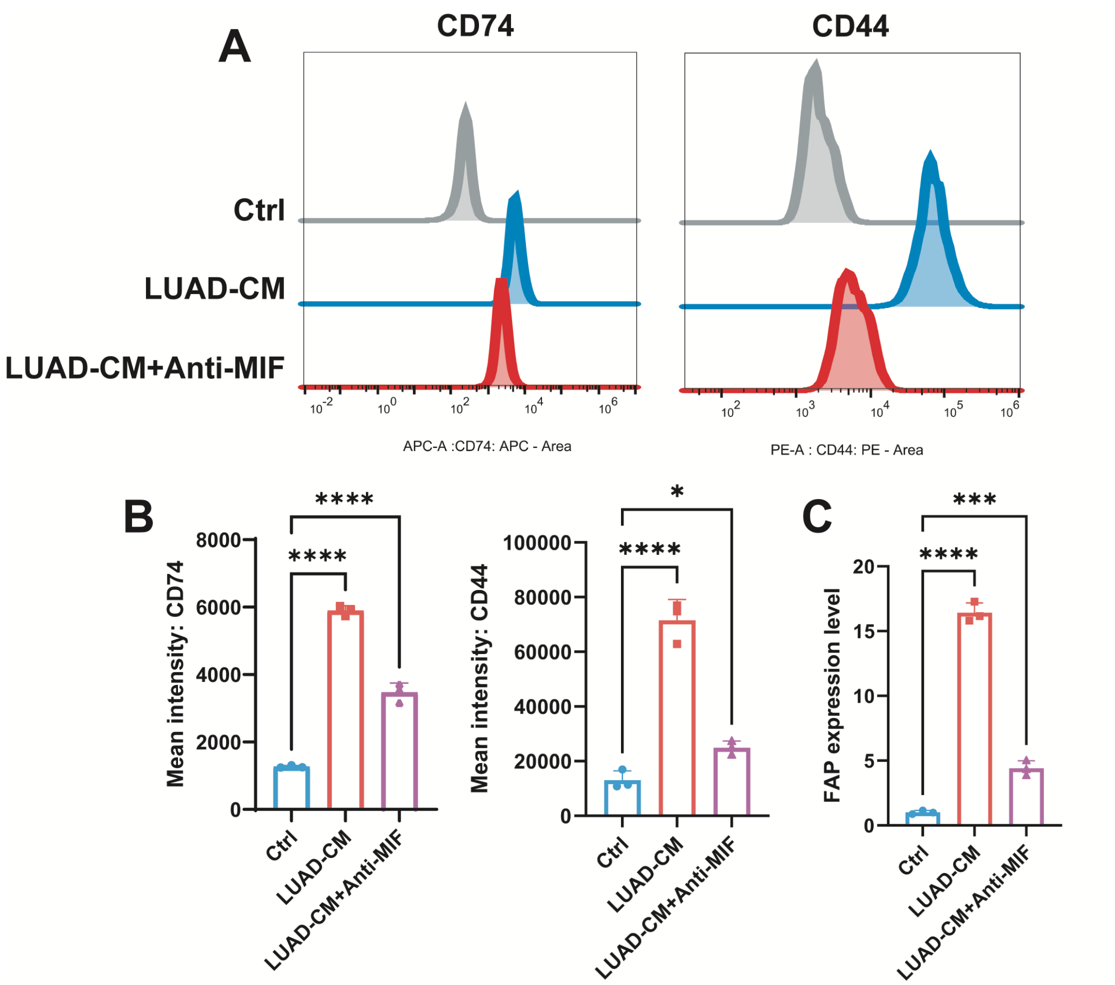

Figure S5. ***In vitro*** **validation of MIF-associated fibroblast activation induced by LUAD-conditioned medium.**

(A) Representative flow cytometry histograms showing CD74 and CD44 expression in HFL1 fibroblasts treated with control medium, LUAD-conditioned medium (LUAD-CM), or LUAD-CM supplemented with a neutralizing anti-MIF antibody.

(B) Quantification of mean fluorescence intensity (MFI) for CD74 and CD44 in fibroblasts across the indicated treatment groups. LUAD-CM increased CD74 and CD44 expression, whereas MIF blockade partially attenuated this induction.

(C) qRT-PCR analysis of FAP expression in fibroblasts treated with control medium, LUAD-CM, or LUAD-CM plus anti-MIF antibody. LUAD-CM markedly increased FAP expression, and this effect was partially reduced by anti-MIF treatment.

Data are presented as mean ± SD from three independent replicates. Statistical significance was determined by one-way ANOVA with Tukey's multiple-comparison test. *$P < 0.05$, ***$P < 0.001$, ****$P < 0.0001$.

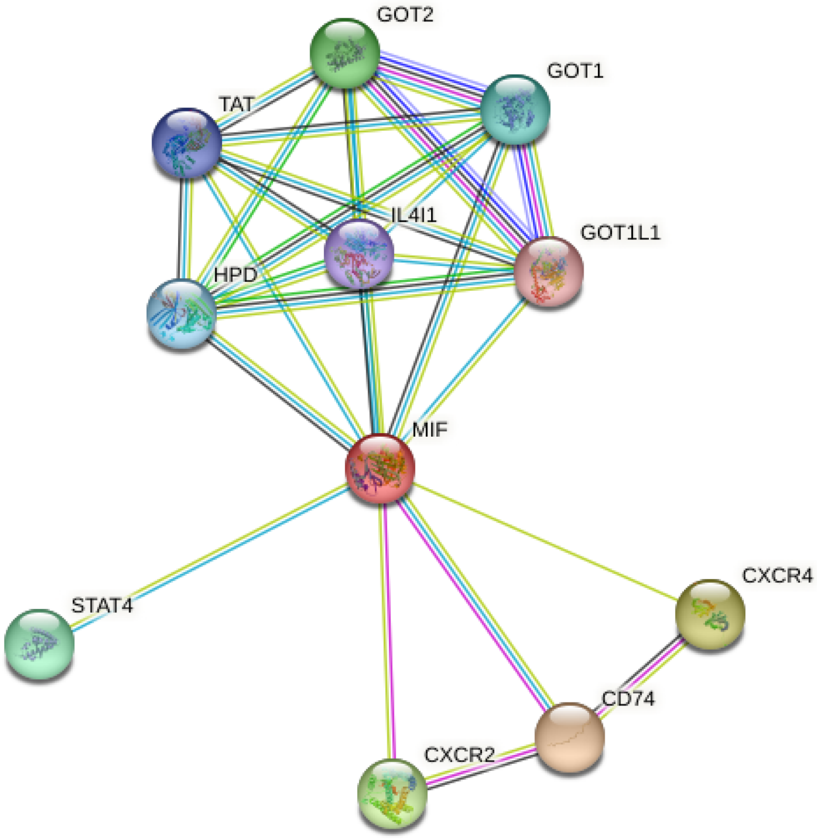

Figure S6. Protein–protein interaction network of MIF-related genes.
Protein–protein interaction network of MIF-related genes constructed using the STRING database.

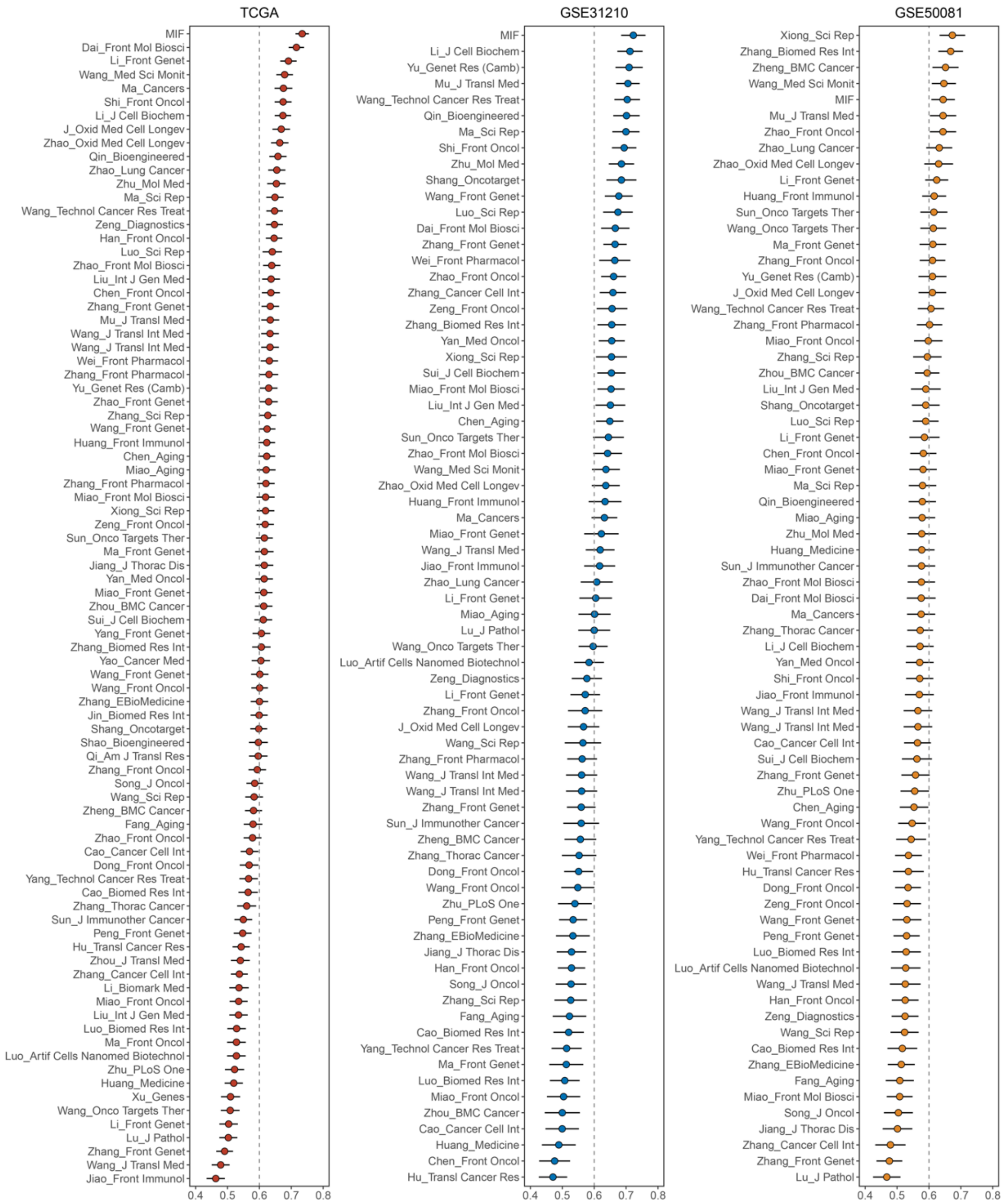

Figure S7. Comparative prognostic performance of the MIF-related signature and published signatures.

C-index comparison of the MIF-related signature and 84 previously published prognostic signatures in the TCGA-LUAD cohort (n = 392), GSE31210 cohort (n = 226), and GSE50081 cohort (n = 127).

Supplementary Table Legend

Table S1. Gene sets used for functional signature scores.

List of genes used to calculate functional signature scores, including cytotoxicity, exhaustion, proliferation, naiveness and Treg-related programs.

Table S2. Raw data for in vitro validation of MIF-associated fibroblast activation.

This table provides the raw experimental data used for Figure S5. The co-culture/conditioned-medium worksheet contains replicate-level flow cytometry measurements of CD74 and CD44 mean fluorescence intensity in HFL1 fibroblasts treated with control medium, LUAD-conditioned medium, or LUAD-conditioned medium plus anti-MIF antibody. The qPCR worksheet contains Ct values for ACTB and FAP, together with the calculated relative FAP expression levels across the indicated treatment groups.
