# Supplementary material for: MIF-Associated Immunosuppressive CAF Remodeling Predicts Poor Prognosis During Lung Adenocarcinoma Progression: A Single-Cell and Multicohort Transcriptomic Study
Source: Biomedicines. 2026 Jul 15;14(7):1581. doi: 10.3390/biomedicines14071581 (PMC13405759; doi:10.3390/biomedicines14071581)
Supplement: Supplementary file 1 [file biomedicines-14-01581-s001.zip › Table S1.pdf]

Table S1. Genes used to calculate signature scores.

Cell type	Signature type	Gene
T cell	Naïveness	*CCR7*
T cell	Naïveness	*TCF7*
T cell	Naïveness	*LEF1*
T cell	Naïveness	*SELL*
T/NK cell	Cytotoxicity	*PRF1*
T/NK cell	Cytotoxicity	*IFNG*
T/NK cell	Cytotoxicity	*GNLY*
T/NK cell	Cytotoxicity	*NKG7*
T/NK cell	Cytotoxicity	*GZMB*
T/NK cell	Cytotoxicity	*GZMA*
T/NK cell	Cytotoxicity	*GZMH*
T/NK cell	Cytotoxicity	*KLRK1*
T/NK cell	Cytotoxicity	*KLRB1*
T/NK cell	Cytotoxicity	*KLRD1*
T/NK cell	Cytotoxicity	*CTSW*
T/NK cell	Cytotoxicity	*CST7*
CD4+ T cell	Exhaustion	*CXCL13*
CD4+ T cell	Exhaustion	*NR3C1*
CD4+ T cell	Exhaustion	*FABP5*
CD4+ T cell	Exhaustion	*RBPJ*
CD4+ T cell	Exhaustion	*ALOX5AP*
CD4+ T cell	Exhaustion	*ID2*
CD4+ T cell	Exhaustion	*ITM2A*
CD4+ T cell	Exhaustion	*FKBP5*
CD4+ T cell	Exhaustion	*DUSP4*
CD4+ T cell	Exhaustion	*LINC01871*
CD4+ T cell	Exhaustion	*RNF19A*
CD4+ T cell	Exhaustion	*CPM*
CD4+ T cell	Exhaustion	*COTL1*
CD4+ T cell	Exhaustion	*ZEB2*
CD8+ T cell	Exhaustion	*CXCL13*
CD8+ T cell	Exhaustion	*CCL3*
CD8+ T cell	Exhaustion	*GZMB*
CD8+ T cell	Exhaustion	*RGS1*
CD8+ T cell	Exhaustion	*ALOX5AP*
CD8+ T cell	Exhaustion	*TNFRSF18*
CD8+ T cell	Exhaustion	*IFNG*
CD8+ T cell	Exhaustion	*TIGIT*
CD8+ T cell	Exhaustion	*RBPJ*
CD8+ T cell	Exhaustion	*ANKRD28*
CD8+ T cell	Exhaustion	*PGAM1*

CD8+ T cell	Exhaustion	*CTLA4*
CD8+ T cell	Exhaustion	*ITM2A*
CD8+ T cell	Exhaustion	*DUSP4*
CD4+ T cell	Treg	*CCL3L1*
CD4+ T cell	Treg	*CD72*
CD4+ T cell	Treg	*CLEC5A*
CD4+ T cell	Treg	*FOXP3*
CD4+ T cell	Treg	*ITGA4*
CD4+ T cell	Treg	*L1CAM*
CD4+ T cell	Treg	*LIPA*
CD4+ T cell	Treg	*LRP1*
CD4+ T cell	Treg	*LRRC42*
CD4+ T cell	Treg	*MARCO*
CD4+ T cell	Treg	*MMP12*
CD4+ T cell	Treg	*MNDA*
CD4+ T cell	Treg	*MRC1*
CD4+ T cell	Treg	*MS4A6A*
CD4+ T cell	Treg	*PELO*
CD4+ T cell	Treg	*PLEK*
CD4+ T cell	Treg	*PRSS23*
CD4+ T cell	Treg	*PTGIR*
CD4+ T cell	Treg	*ST8SIA4*
CD4+ T cell	Treg	*STAB1*
T/NK cell	Proliferation	*ZWINT*
T/NK cell	Proliferation	*E2F1*
T/NK cell	Proliferation	*FEN1*
T/NK cell	Proliferation	*FOXM1*
T/NK cell	Proliferation	*H2AFZ*
T/NK cell	Proliferation	*HMGB2*
T/NK cell	Proliferation	*MCM2*
T/NK cell	Proliferation	*MCM3*
T/NK cell	Proliferation	*MCM4*
T/NK cell	Proliferation	*MCM5*
T/NK cell	Proliferation	*MCM6*
T/NK cell	Proliferation	*MKI67*
T/NK cell	Proliferation	*MYBL2*
T/NK cell	Proliferation	*PCNA*
T/NK cell	Proliferation	*PLK1*
T/NK cell	Proliferation	*CCND1*
T/NK cell	Proliferation	*AURKA*
T/NK cell	Proliferation	*BUB1*
T/NK cell	Proliferation	*TOP2A*
T/NK cell	Proliferation	*TYMS*
T/NK cell	Proliferation	*DEK*

T/NK cell	Proliferation	*CCNB1*
T/NK cell	Proliferation	*CCNE1*
